# Supplementary material for: Can welfare states buffer technostress? Income and technostress in the context of various OECD countries
Source: PLoS One. 2023 Dec 5;18(12):e0295229. doi: 10.1371/journal.pone.0295229 (PMC10697519; doi:10.1371/journal.pone.0295229)
Supplement: Attachment — Submitted filename: Response to Reviewers PONE-D-22-33306_R1_ 2023-06-24.docx [file pone.0295229.s001.docx]

# **Summary of Major Changes**

We would first like to express our sincere thanks to Dr. Cowley and our reviewer for the valuable comments and suggestions we received after our initial submission. We appreciated that you felt confident enough in our research to allow us to revise and resubmit our work. Your comments and feedback were insightful and developmental. Thank you for taking the time.

We have worked through the reviews and made substantial revisions to the manuscript to respond to your comments and suggestions. We would like to summarize the main adjustments here:

(1) First, we followed the feedback regarding the theoretical bridge between income and technostress by adding two insightful paragraphs on the relationship between income, job insecurity, and technostress (p. 7-8).
(2) We also addressed all the methodological concerns raised by the reviewer and provide now, for example, a detailed explanation of our logged income variable (p. 12). In addition, we clarified the order of the results section (p. 17).

Finally, we made several other minor changes to the manuscript, which are explained in detail in the following sections, when answering your specific requests point by point.

# **Editorial Comments**

***Comment #0****: Dear Dr. Lauterbach,*

*Thank you for submitting your manuscript to PLOS ONE. After careful consideration, we feel that it has merit but does not fully meet PLOS ONE’s publication criteria as it currently stands. Therefore, we invite you to submit a revised version of the manuscript that addresses the points raised during the review process.*

*Please note that we have only been able to secure a single reviewer to assess your manuscript. We are issuing a decision on your manuscript at this point to prevent further delays in the evaluation of your manuscript. Please be aware that the editor who handles your revised manuscript might find it necessary to invite additional reviewers to assess this work once the revised manuscript is submitted. However, we will aim to proceed on the basis of this single review if possible.*

*While overall positive towards publication, the reviewer has provided some comments regarding opportunities for clarification and elaboration. Please ensure you address each of the reviewer's comments when revising your manuscript.*

**Our answer:** Thank you for your confidence in our work and the opportunity to revise and resubmit. We will do our best to address your and the reviewer's concerns thoroughly.

***Comment #1****: Please ensure that your manuscript meets PLOS ONE's style requirements, including those for file naming.*

**Our answer:** We have had the entire draft professionally checked for the style requirements.

***Comment #2****: Peer review at PLOS ONE is not double-blinded (https://journals.plos.org/plosone/s/editorial-and-peer-review-process). For this reason, authors should include in the revised manuscript all the information removed for blind review.*

**Our answer:** Thank you for this remark. We included the previously anonymized references.

***Comment #3****: Thank you for stating the following financial disclosure: “The authors acknowledge the funding by the Deutsche Forschungsgemeinschaft (DFG—German Research Foundation) under Germany’s Excellence Strategy (Grant Number EXC2035/1-390681379).”*

*Please state what role the funders took in the study. If the funders had no role, please state: "The funders had no role in study design, data collection and analysis, decision to publish, or preparation of the manuscript." If this statement is not correct you must amend it as needed. Please include this amended Role of Funder statement in your cover letter; we will change the online submission form on your behalf.*

**Our answer:** Thank you for pointing this out. We added the statement in our cover letter.

***Comment #4:*** *Please amend your authorship list in your manuscript file to include author names.*

**Our answer:** Thank you for this remark. We amended the author names and institutions in the manuscript.

# **Comments Reviewer 1**

## 1. Is the manuscript technically sound, and do the data support the conclusions?

***Comment #1:*** Yes

**Our answer:** Thank you for your positive feedback.

## 2. Has the statistical analysis been performed appropriately and rigorously?

***Comment #2:*** Yes

**Our answer:** Thank you for the encouragement of our method.

## 3. Have the authors made all data underlying the findings in their manuscript fully available?

***Comment #3:*** Yes

**Our answer:** Thank you for acknowledging our data access.

# 4. Is the manuscript presented in an intelligible fashion and written in standard English?

***Comment #4:*** Yes

**Our answer:** Again, we thank you for the affirmation that we have created a sound contribution.

# 5. Review Comments to the Author

***Comment #5.0:*** This is a well written and executed study on income, technostress, and welfare state protection. It will make a nice contribution to the literature. I have a few minor points the authors may want to consider in their revision.

**Our answer:** Thank you very much for your detailed feedback. We will address all of your specific concerns thoroughly below.

***Comment #5.1:*** Until I got to the methods section where the measure of technostress is described, I was wondering where job insecurity fits into all of this. I think it would be helpful to add a paragraph or two that describes the relationship between job insecurity and technostress.

**Our answer:** Thank you for this remark on the theoretical framework. We agree that our argumentation for selecting job insecurity as a mechanism was too shortly explained in the theory part of the prior version. Therefore we added more details on this in the hypotheses development (pp. 7-8) section.

***Comment #5.2:*** It would be helpful if you included the actual scale that the individual components of the technostress latent variable were measure on.

**Our answer:** We added the original scale to Fig 1.

***Comment #5.3:*** It may be worth noting why income is logged.

**Our answer:** Thank you for this comment. Based on your recommendation, we added a footnote (new Footnote 2) explaining the rationale for logging income.

***Comment #5.4:*** I found it a bit odd that in the Results section the authors began with a discussion of the findings of the control variables and then the discussion of the findings relevant to the research questions followed. I would consider switching this.

**Our answer:** Thank you for this remark. We changed the order of the results section to improve the reader’s understanding.

Finally, we want to thank you again for the diligent review of our paper and the concrete ideas on how to improve it. We hope that you generally appreciate our revision.
