# Supplementary material for: Can welfare states buffer technostress? Income and technostress in the context of various OECD countries
Source: PLoS One. 2023 Dec 5;18(12):e0295229. doi: 10.1371/journal.pone.0295229 (PMC10697519; doi:10.1371/journal.pone.0295229)
Supplement: Attachment — Submitted filename: Review report.docx [file pone.0295229.s002.docx]

**Review report**

The study reveals that the authors aimed to explore the influence of the welfare state context on the correlation between income and technostress. Nevertheless, the study could benefit from a restructuring. The introduction section would benefit from the inclusion of research motivation and the identification of research gaps. The theory section lacks in-depth discussion. Notably, the stress appraisal theory does not align with some of the proposed relationships in the study, such as that between income and technostress. Furthermore, the discussion in the hypothesis development section diverges from the proposed hypotheses. It would be advisable for the authors to reconsider the research model in accordance with a more appropriate theory. Enhancements are also needed in the research methodology section, particularly regarding insights into the data collection process, respondent profiles, and the criteria employed for respondent selection.

Moreover, the study indicates the utilization of both primary and secondary data. The author could present robust arguments, accompanied by citations, demonstrating the suitability of utilizing data gathered from both primary and secondary sources for constructing a research model. However, there appears to be a lack of clarity regarding the source of measurement for the income variable. Additionally, it is advisable for the control variables to be substantiated by referencing prior literature. The statistical techniques employed should be properly justified in relation to the collected data. Finally, there is room for enhancement in the sections covering results, discussion, and conclusions.
