# Supplementary material for: Can welfare states buffer technostress? Income and technostress in the context of various OECD countries
Source: PLoS One. 2023 Dec 5;18(12):e0295229. doi: 10.1371/journal.pone.0295229 (PMC10697519; doi:10.1371/journal.pone.0295229)
Supplement: Attachment — Submitted filename: Response to Reviewers PONE-D-22-33306_R2_ 2023-10-04.docx [file pone.0295229.s003.docx]

# **Summary of Major Changes**

We would like to express our sincere thanks to Prof. Luqman and our two reviewers for the valuable comments and suggestions we received after the first round of review. We appreciate that you felt confident enough in our research to allow us to revise and resubmit our work again. Your comments and feedback were insightful and developmental. Thank you for taking the time.

We have worked through the reviews and made substantial revisions to the manuscript to respond to your comments and suggestions. We want to summarize the main adjustments here:

(1) First, we followed the feedback regarding the restructuring and readability of our study by a) stating a more detailed research motivation (p. 4), b) providing more context and literature to embed the hypothesis development in our theoretical stress framework (e.g., p. 7-9), and c) delving deeper into the discussion and comparing our results to previous research (p. 22-23).
(2) We also addressed all the methodological concerns raised by reviewer 2 and provide now, for example, more details on our data (p. 13) and the reasoning behind the choice of certain control variables (p. 16). Further, we explained why the hierarchical Item Response Theory (IRT) model allows us to combine our individual-level data with information on the country level (p. 17).

Finally, we made several other minor changes to the manuscript, which are explained in detail in the following sections, when answering your specific requests point by point.

# **Editorial Comments**

***Comment #0****: Dear Dr. Lauterbach,*

*Thank you for submitting your manuscript to PLOS ONE. After careful consideration, we feel that it has merit but does not fully meet PLOS ONE’s publication criteria as it currently stands. Therefore, we invite you to submit a revised version of the manuscript that addresses the points raised during the review process.*

*While overall positive towards publication, the reviewer has provided some comments regarding opportunities for clarification and elaboration. Please ensure you address each of the reviewer's comments when revising your manuscript.*

**Our answer:** First, thank you for taking on the editor role for our manuscript. We appreciate your confidence in our work. We were glad that reviewer one was content with our first revised version. We will do our best to address the second reviewer's concerns thoroughly.

***Comment #1****: While revising your submission, please upload your figure files to the Preflight Analysis and Conversion Engine (PACE) digital diagnostic tool,*

[*https://pacev2.apexcovantage.com/*](https://pacev2.apexcovantage.com/)*. PACE helps ensure that figures meet PLOS requirements.*

**Our answer:** Thank you for this note, we uploaded all figures and made changes where applicable. The figures in the appendix are updated. We could also offer to upload the tif-formatted graphs separately.

# **Comments Reviewer 1**

***Comment #1:*** *All comments have been addressed. The authors have done a nice job addressing my comments. I look forward to seeing it in publication.*

**Our answer:** Thank you for your positive feedback – we were glad to see that you acknowledged our changes based on the previous round of review and feel like our manuscript is now acceptable for publication. We look forward to seeing it in publication, too!

# **Comments Reviewer 2**

***Comment #1:*** *The study reveals that the authors aimed to explore the influence of the welfare state context on the correlation between income and technostress. Nevertheless, the study could benefit from a restructuring.*

**Our answer:** Thank you very much for your detailed feedback. We will address all of your specific concerns thoroughly below.

***Comment #2:*** *The introduction section would benefit from the inclusion of research motivation and the identification of research gaps*.

**Our answer:** Thank you for pointing this out. We revised the introduction section and ensured the research motivation is now more nuanced than in the previous version. In particular, as you can see on page 4, we highlighted that previous research examining the income-technostress relationship focused on particular technostress facets (e.g., technoinvasion) and, thus, neglected the multifaceted nature of technostress perceptions. In addition, we strengthened the point of combining the micro- and macro-level perspectives in our study (p. 5-6) through our comparative study on an cross-national level, which we see as our main theoretical contribution.

***Comment #3:*** *The theory section lacks in-depth discussion. Notably, the stress appraisal theory does not align with some of the proposed relationships in the study, such as that between income and technostress. Furthermore, the discussion in the hypothesis development section diverges from the proposed hypotheses. It would be advisable for the authors to reconsider the research model in accordance with a more appropriate theory.*

**Our answer:** Thank you for this remark on the theoretical framework. We agree that our argumentation for selecting the stress appraisal theory to develop our hypothesis was too shortly explained in the prior version. Therefore, we made several changes to clarify the relationship between income and technostress and embedded it more in the theoretical framework, e.g., on page 7, where we explain that "*the individual response to such stressors might depend on a worker’s life circumstances and the respective available resources*." Furthermore, we restructured the hypothesis development section regarding the income and technostress relationship, providing the reader with less complicated sentences and a better reading flow (see p. 7-9).

***Comment #4:*** *Enhancements are also needed in the research methodology section, particularly regarding insights into the data collection process, respondent profiles, and the criteria employed for respondent selection.*

**Our answer:** To clarify these aspects, we added more detailed information on the Risks that Matter survey, in particular, who conducted the survey, how it was implemented, and which quotas were used for the sampling procedure (see p. 13).

***Comment #5:*** *Moreover, the study indicates the utilization of both primary and secondary data. The author could present robust arguments, accompanied by citations, demonstrating the suitability of utilizing data gathered from both primary and secondary sources for constructing a research model.*

**Our answer:** Thank you for this note. We provided more detailed insights into our primary and secondary data (see p. 13). It is common practice in hierarchical modeling focusing on individuals nested in countries that the first-level (individual) data comes from a different data source than the second-level (country) data. While country-level data is often readily available, individual-level data is more challenging to come by, particularly in the social sciences where established surveys like the European Social Survey (ESS) or the International Social Survey Programme (ISSP) cover only a limited amount of topics. Thus, recent years have seen an explosion of social science survey projects in which researchers conduct their own surveys to explore topics of their own choosing. Our individual-level data is an example of that but in a hybrid form, as the data were collected as part of an established survey of the OECD. For the first time in existing research, this cooperation allowed us to ask questions about technology-related risks to individuals from a broad range of country backgrounds. All the individual-level data we use come from the OECD Risks that Matter survey (primary data source), and only the country-level data come from other sources (secondary data sources), the latter being explicitly modeled differently in the hierarchical model. We tried to be more explicit about this in the revised version of the paper.

***Comment #6:*** *There appears to be a lack of clarity regarding the source of measurement for the income variable. Additionally, it is advisable for the control variables to be substantiated by referencing prior literature.*

**Our answer:** Closely related to the previous comment, the question on the source of the income variable makes it clear to us that we need to be clearer about the RTM survey being the sole source of the individual-level data. We thank the reviewer for pointing this out to us and have attempted to correct this shortcoming in the revised version. In addition, we added references to justify the selection of control variables on the individual level. As we are – to our knowledge – the first to also consider the potential impact of country factors on technostress, there is no literature we can draw on. Thus, we added more detailed explanations for why we think that these controls are reasonable.

***Comment #7:*** *The statistical techniques employed should be properly justified in relation to the collected data.*

**Our answer:** Thank you for pointing this out. We added more explanation for how our statistical model relates to the data (please see p. 16-17), explaining why the hierarchical IRT model allows us to combine our individual-level data with information on the country level.

***Comment #8:*** *Finally, there is room for enhancement in the sections covering results, discussion, and conclusions.*

**Our answer:** Thank you for this remark. We revised all these sections to improve the reader’s understanding. In particular, we added an introductory paragraph at the beginning of the result section (p. 18-19). Regarding the discussion, we agree that discussing our findings with previous research was possible. Therefore, we added various comparisons to existing research, especially for the income-technostress relationship (p. 22-23).

Finally, thank you again for the diligent review of our paper and the concrete ideas for improving it. We really delved deep into our manuscript again and present it now more intelligibly, with improved readability and methodological rigor. We hope that you generally appreciate our revision and that it brought us closer to a publication in PLOS ONE.
