# Supplementary material for: Can welfare states buffer technostress? Income and technostress in the context of various OECD countries
Source: PLoS One. 2023 Dec 5;18(12):e0295229. doi: 10.1371/journal.pone.0295229 (PMC10697519; doi:10.1371/journal.pone.0295229)
Supplement: Attachment — Submitted filename: Response to Reviewers PONE-D-22-33306_R23_ 2023-11-10.docx [file pone.0295229.s004.docx]

# **Summary of Major Changes**

We would like to express our sincere thanks to Prof. Luqman and our reviewers for the valuable comments and suggestions we received after the second round of review. We appreciate that you felt confident enough in our research to allow us to revise and resubmit our work again. Thank you for taking the time.

We have worked through the reviews and made several changes to the manuscript, which are explained in detail in the following sections, when answering your specific requests point by point.

# **Editorial Comments**

***Comment #1:*** *Please review your reference list to ensure that it is complete and correct. If you have cited papers that have been retracted, please include the rationale for doing so in the manuscript text, or remove these references and replace them with relevant current references. Any changes to the reference list should be mentioned in the rebuttal letter that accompanies your revised manuscript. If you need to cite a retracted article, indicate the article’s retracted status in the References list and also include a citation and full reference for the retraction notice.*

**Our answer:** Thank you for pointing this out. We meticulously reviewed our reference list and confirmed that no article was retracted using the website <http://retractiondatabase.org/RetractionSearch.aspx>

Regarding new references, we included the following references to support our revised version:

Fetscher, Verena (2023). Explaining support for redistribution: social insurance systems and fairness. *Political Science Research and Methods*, *11*, 588-604.

Nisafani, A. S., Kiely, G., & Mahony, C. (2020). Workers’ technostress: A review of its causes, strains, inhibitors, and impacts. *Journal of Decision Systems*, *29*(1), 243-258.

OECD (2021). Main Findings from the 2020 Risks that Matter Survey, OECD Publishing, Paris.

Scruggs, Lyle A. and Gabriela Ramalho Tafoya (2022). Fifty years of welfare state generosity. *Social Policy & Administration, 56*(5), 791-807.

# **Comments Reviewer 2**

***Comment #1:*** *Thank you for re-inviting me to review the manuscript entitled "Can welfare states buffer technostress, Income and technostress in the context of various OECD countries". The author made a good effort. However, I still want to the author to consider the following.*

**Our answer:** Thank you very much for your detailed feedback. We will address all of your specific concerns thoroughly below.

***Comment #2:*** *Lines 8 and 9 in the "income and technostress" section appear to be confusing. To clarify, please provide an explanation supported by scholarly citations or a logical argument regarding the role of income in the first and second appraisal of stress.*

**Our answer:** Thank you for pointing this out. We carefully revised and slightly restructured the relevant section in order to bring more clarity to our argumentation. We now start the argument with the following statement (p. 7f):

*“ … higher-income individuals might identify technological stressors in the primary appraisal of stress but also see their financial resources as a valuable tool to cope with these stressors. Therefore, the secondary appraisal of whether there are enough resources available to overcome difficulties with technological stress (e.g., feeling overwhelmed at work and fear of job loss) turns out optimistic.”*

This theoretical presentation is followed by the argumentation about job insecurity and technostress as before. Finally, there are two sections on the empirical evidence to date (p. 9f): first on socioeconomic status and technostress in general, then more specifically on income and stress or technostress. In this way, the current evidence is emphasized more clearly with the appropriate references.

***Comment #3:*** *Please fortify hypothesis 2b with appropriate scholarly citations.*

**Our answer:** Thank you for this remark on hypothesis 2b. Since the proposed cross-level interactive relationship is -- to our best knowledge -- novel, there is no authoritative literature that we could draw on. In the revised version, we, therefore highlight the novelty of the argument and calarify that the hypothesis is introduced by us (p. 12). Moreover, we added an recently published reference, which shows empirically that high-income earners are more supportive of insurance designs if the benefits are provided in relation to previous income, as this finding reinforces the underlying mechanism of our proposed hypothesis.

***Comment #4:*** *Provide a more detailed explanation of quota sampling, including the population proportion allocated to each quota. In case uncontrolled quota sampling is used, be sure to indicate this in the study limitations.*

**Our answer:** To clarify these aspects, we added more information on the quota sampling, which is based on representative population data for each country. We also included a reference to the main OECD report that provides a description of the sampling procedure (see p.13).

***Comment #5:*** *Clearly state which indicators were utilized to measure "Welfare State Generosity" and explain how these indicators were managed in the construction of the "Welfare State Generosity" scale.*

**Our answer:** Thank you for this note. In the revised version of the paper, we state more clearly that welfare state generosity is measured by a country's unemployment benefits (net replacement rates of previous income). We also added a reference (Scruggs & Gabriela, 2022) that underscores that this is a common measure of welfare state generosity in the literature (p. 15).

***Comment #6:*** *Address how missing data for Chile and Mexico were handled during the analysis.*

**Our answer:** Thank you for pointing this out. Since data for Chile and Mexico are not available in our dataset, these two countries are not included in the analysis. The revised version of the paper states this more explicitly (p. 15).

***Comment #7:*** *Consider separating the "implications" section from the "discussion and conclusion" section.*

**Our answer:** We agree that the final section of our paper could still be better structured. Therefore, we divided the section into two main sections “discussion” and “conclusion”, whereby “discussion” was divided into subsections including the summary of the main findings, theoretical and practical implications, and limitions and future research. In addition, we reordered the sections according to these new headings (see p. 23-25).

***Comment #8:*** *Evaluate if the study carries theoretical implications and, if so, include them in the paper following the discussion section.*

**Our answer:** Thank you for this remark. We revised the implications part of the discussion section and clarified where we see our theoretical contribution. We emphasize that our study is the first multilevel perspective on the technostress research field, which has so far focused strongly on the individual and organizational level (p. 24, paragraph 1). Further, we stress that technostress has so far received too little attention in comparative welfare state research - although such stress is increasingly becoming the norm due to advancing technological developments in the work environment (p. 24, paragraph 2).

Finally, thank you again for the diligent and patient review of our paper and the concrete ideas for improving it. We really feel confident about our current version and hope that it brought us closer to a publication in PLOS ONE.
